# A Peculiar Case

**Published:** 1891-11

**Authors:** 


### EDITORIAL.

### A Peculiar Case.

Mrs. P——, about two years ago had a left inferior first molar crowned—gold crowned. The tooth was so much decayed at the time of the operation, that this course was thought preferable to any attempt to fill it. The pulp still living, not exposed. The dentine in the cavities of decay somewhat sensitive. The gold crown being set had such contact with the upper molar, that it was necessary to dress it, and doing this, at two points the crown was cut through, and the cement beneath exposed at only a small surface. Repair was not then made of these points, but they were permitted to remain with the cement exposed; and either for want of attention on the part of the patient or the dentist, the case has gone along in this condition for the time above specified; the openings through the gold meanwhile becoming considerably larger by wear. During the last two or three months there has been an increasing sensitiveness of the tooth in mastication, so that, for the last six weeks or more, it has been impossible to masticate upon the tooth. There was no periosteal trouble, striking upon the tooth causing no pain, but the moment any attempt at mastication upon the tooth was attempted, unbearable pain occurred. The cement seemed apparently in good condition at the openings. The annoyance became so great that the removal of the crown was decided upon. This was done a few days ago. The crown was very firmly in its position, so that it was cut open on one side, and simply stripped off of the tooth and the cement which held it in its position; this being done, examination with the instrument revealed the fact that the cement opposite the openings of the crown was somewhat softened, though not wholly disintegrated, so that moisture penetrated it, and so it afforded but little if any resistance to pressure upon it. Upon removing this it was found that the dentine beneath was beginning to decompose, and was exceedingly sensitive, so that the mere contact of the instrument with the surface caused excrutiating pain. A few thrusts of the sharp excavator cutting at this affected portion, almost entirely relieved the sensitiveness; and when all the dentine affected by decay was removed, there was scarcely more than the normal sensitiveness of the dentine. The exposed surface was thoroughly dried, carbolic acid applied, it was then covered with a hard sticky wax, and the patient dismissed. An examination after twenty-four hours revealed the fact that there was scarcely a vestige of return of the former trouble, and masticating upon that side gave no pain, though the patient was somewhat timid about it. This case had been examined by two or three dentists without any conclusions as to the cause of the trouble, indeed, it did appear obscure, but after going to the bottom of the matter, it was very clear. By the influence of moisture and mastication, the cement inside of these little openings, had softened enough to admit moisture and the chill of cold fluids, which caused great pain, and sufficient movement of the cement to irritate the sensitive dentine beneath.

Now, the teaching of all this is, never permit an opening through a gold crown upon a tooth or root having a live pulp, though it may apparently be secured by the cement within the cap; usually, if such a little opening is made, by dressing for proper occlusion the breach can be repaired by simply dressing out enough to receive a little gold filling, in that way such openings can be effectually stopped. Another lesson is taught by this case, never rest satisfied with an obscure case without making thorough examination. It is well to attempt very little treatment, and in most cases none at all, when one cannot reach to the bottom of obscure cases.

